# Effects of acupressure on sleep quality and anxiety of patients with second- or third-degree burns: a randomized sham-controlled trial

**DOI:** 10.1186/s12906-023-04292-2

**Published:** 2024-01-02

**Authors:** Zahra Salajegheh, Mehdi Harorani, Mohadese Shahrodi, Elahe Dolati, Mahtab Farahani, Nazanin Amini, Danial Habibi

**Affiliations:** 1 Department of Medical-Surgical Nursing, School of Nursing and Midwifery, Kerman University of Medical Sciences, Kerman, Iran; 2 Department of Nursing, Shazand School of Nursing, Arak University of Medical Sciences, Arak, Iran; 3 Traditional and Complementary Medicine Research Center (TCMRC), Arak University of Medical Sciences, Arak, Iran; 4 Department of Intensive Care Nursing, Gonbad Kavoos Branch, Islamic Azad University, Gonbad Kavoos, Iran; 5 Department of Operating Room, Azadshahr Branch, Islamic Azad University, Azadshahr, Iran; 6 Student Research Committee, Department of Nursing, School of Nursing, Arak University of Medical Sciences, Arak, Iran; 7 School of Paramedicine, Arak University of Medical Sciences, Arak, Iran; 8 Department of Biostatistics and Epidemiology, School of Health, Isfahan University of Medical Sciences, Isfahan, Iran

**Keywords:** Acupressure, Anxiety, Burn, Sleep Hygiene

## Abstract

**Background:**

Although acupressure is proposed to boost sleep quality and alleviate anxiety in various disorders, no trials have yet documented these consequences in burn victims. Considering the high importance of managing sleep quality and anxiety among burn patients utilizing adjunctive non-pharmacological measures, this study sought to investigate the impacts of acupressure on sleep quality and anxiety among a population of Iranian patients with burn injuries.

**Methods:**

This trial was performed on 72 patients with second- or third-degree burns, who were divided into two equal arms to receive routine care plus 10-minute acupressure on either real acupoints (i.e., *Yintang* and *Shen men*) or sham points for three consecutive nights. Sleep quality and anxiety were investigated at baseline (T1) and on the fourth day (T2) by using St. Mary’s Hospital Sleep Questionnaire (SMHSQ) and Spielberger’s State-Trait Anxiety Inventory for State Anxiety (STAI-S), respectively.

**Results:**

The mean scores of SMHSQ and STAI-S were significantly lower in the real acupressure arm at T2 (*P <* 0.001 in two cases), implying better sleep quality improvement and higher anxiety alleviation. Also, the reduction in mean changes of SMHSQ and STAI-S scores from T1 to T2 was significantly more in the real acupressure arm (*P <* 0.001 in two cases).

**Conclusion:**

Acupressure, as a low-cost complementary method, could be potentially helpful in enhancing sleep quality and decreasing the anxiety of burn patients. Additional long-term trials are required to identify the sustainability of the findings.

**Trial Registration No:**

IRCT20130424013110N13 (Registration date: 19/03/2021, https://www.irct.ir/trial/55076).

## Background

Burn is a prominent global health-related concern and one of the most critical traumas resulting in hospitalization and substantial morbidity and mortality [1]. According to available data, approximately 180,000 individuals die annually from burn-related complications [2]. Although the mortality rate and incidence of burns have declined considerably in developed societies, burn-related injuries are still a common health challenge in low- and middle-income regions [3]. In Iran, a large country in the Middle East, the incidence of different types of burns is demonstrated to be still high [4–6]. Besides, burn damages are even a notable cause of death in Iran among all age groups [7], and Iranian burn patients’ mortality rate is 10–20% [8, 9].

Following burns, patients experience different physiological problems (e.g., contractures, scarring, infection, chronic inflammation, pain, and hypothermia). Besides, behavioral and psychological complications are prevalent among burn survivors, including but not limited to post-traumatic stress disorder, personality changes, phobias, depression, anxiety, and sleep disturbances [10]. Among these, anxiety is a common annoying problem, often due to patient’s concerns about their appearance, the painful nature of therapeutic procedures (i.e., wound debridement, wound dressing change, and repair surgeries), and monetary and occupational problems [11–13]. In addition to high anxiety, recent surveys documented poor perceived sleep quality following burn injuries, which could be experienced mainly due to pain, narrow airway, pruritus, and scar appearance during different stages of the recovery process [14–16]. Studies also show a bivariate relationship between anxiety and sleep disturbances among burn sufferers so that high burn-induced anxiety leads to insomnia and poor sleep quality; sleep deprivation also triggers or exacerbates anxiety [17]. These two complications can cause many consequences, such as low commitment to self-care and collaboration with the therapeutic staff, impaired immune system function, delayed wound healing, sympathetic nervous system stimulation, and augmented secretion of stress hormones, followed by raised blood pressure and heart rate [18–20]. Therefore, alleviating anxiety and improving sleep quality among burn victims must be considered to facilitate their complete rehabilitation.

Sedatives and opioids are the most important drugs used in burn care to manage patients’ complications, such as anxiety and sleep disturbances [21]. Although these medications are highly effective for managing burn injury-related problems, they are sometimes associated with side effects and are not tolerated by all patients [22]. Opioid administration after a burn injury can lead to opioid dependence/abuse and other potential consequences [23]. Based on a cohort study, burn patients who experienced opioid use disorder had a significantly higher incidence of mental health disorders (i.e., major depressive, post-traumatic stress, and generalized anxiety), suicidal/homicidal ideations and suicide attempts, and polysubstance abuse, as well as were more likely to use psychiatric services and psychotherapy [24]. Also, 30-day all-cause readmission rates and in-hospital resource utilization were higher among burn patients with opioid dependence, causing a substantial healthcare economic burden [25]. Hence, burn patients should be offered non-pharmacological practices for controlling their complications, at least as adjunctive measures to their standard care program [21].

Many complementary and alternative modalities have been proposed in recent years to relieve anxiety and sleep disturbances following burn injuries, including aromatherapy, relaxation therapy, and massage therapy [17, 26–28]. Acupressure is another non-pharmacological method that has attracted considerable attention in complementary and alternative medicine for relieving sleep and anxiety problems, as it is cost-effective, can be used in almost all patients, and has lower adverse effects compared to pharmacological agents [29, 30]. This technique is rooted in acupuncture and is one of the main parts of traditional Chinese medicine [31]. In this method, acupuncture points (or acupoints) are stimulated by palms, fingers, or devices with specific techniques to control and balance body energy, support good health, and prevent sickness [32].

In recent trials, acupressure utility for managing sleep and anxiety disturbances has been evaluated in different populations. Some studies confirmed the sleep-enhancing effect of acupressure among nursing staff [33] and patients with diabetes [34], as well as those undergoing hemodialysis [35] and cholecystectomy [36]. Also, acupressure reduced anxiety among surgery candidates [37, 38] and cancer patients undergoing chemotherapy [39]. Recent reviews also indicated acupressure efficacy on either sleep quality [40], anxiety [41], or both [29]. Still, they emphasized conducting more rigorous trials among different populations to better elucidate the acupressure effectiveness in these outcomes.

To the best of our knowledge, no trial has yet evaluated the acupressure’s effectiveness in reducing sleep and anxiety problems caused by burns. Yet, a pilot trial documented the utility of acupressure on the *Yintang* (EX-HN 3, third eye) and *Shen men* acupoints for reducing post-dressing pain in individuals suffering from burn injuries [42]. Considering the potential anxiolytic and sleep-enhancing effects of acupressure in other conditions, determining whether these consequences are similar in burn victims is of merit. Hence, based on the pilot trial mentioned above, this trial sought to compare the impacts of acupressure administration in real acupoints (i.e., *Yintang* and *Shen men*) or sham points on burn patients’ sleep quality and anxiety. We hypothesized that, after the interventions, the sleep quality of patients who experienced real acupressure would be higher than those who received sham acupressure. Additionally, we assumed that the experimental arm’s anxiety level would be lower than the control arm at the end of the interventions.

## Methods

### Study design

This was a single-center, randomized, sham-controlled trial with a parallel group design of 72 patients, enrolled from April 2021 to January 2022.

### Setting and participants

This trial was performed on hospitalized patients at the Burn Unit of Valiasr Hospital, Arak, Iran. The eligible participants were included if they: (1) aged 18 to 55 years, (2) had passed the burn emergency stage (i.e., 48–72 h after the occurrence of injury), (3) suffered from unintentional second- or third-degree burns with an area of 15–65%, and (4) had the stability of hemodynamic status. Exclusion criteria were regarded as follows: (1) having burn injuries in/near the determined acupressure points (i.e., head and face), (2) having a history of psychological disorders (i.e., anxiety and stress), sleep disorders, and sleep-disturbing illnesses (i.e., migraine and rheumatoid arthritis), (3) taking anxiolytics, hypnotic-sedative drugs, or opioids in the past 12 h, (4) having an addiction to alcoholic beverages or drugs, and (5) the incidence of critical conditions (e.g., a decline in consciousness level, hemodynamic instability, the need for pulmonary intubation, and hemostasis defect). Likewise, the participants volunteered to remove from the trial, and those who transferred to other clinical wards or had died during the study were excluded.

### Sampling and randomization

The participants were chosen by a convenient sampling strategy. If they were qualified to partake in the study, they were randomly placed in one of the experimental (n = 36) or control (n = 36) groups. Randomization was performed through a block randomization process with an allocation ratio of 1:1 to establish a balance between the groups and prevent selection bias. To this end, 18 quadrupled blocks were generated. Consequently, 36 codes of E for the experimental arm and 36 codes of C for the control arm were noted on paper and put inside concealed envelopes. Considering the block size of four, six combinations of group assignments were possible (i.e., EECC, ECEC, CEEC, CECE, CCEE, and ECCE). Patients selected one envelope during recruitment and were assigned to groups according to the chosen envelope code. The principal investigator conducted the randomization process, and all related information was maintained confidential until the trial’s end.

### Blinding

The patients could not be blinded entirely to the nature of the acupressure. However, to diminish bias, sham acupressure in the non-effective points was presented in the control arm, considering a similar gesture of the intervention, the same amount of time, and the same time points to the experimental arm. Also, participants in the control arm were unaware that acupressure was conducted on sham points. Furthermore, to avoid contact between the study groups, the participants of the experimental and control arms were hospitalized in different rooms. Besides, the nursing assistants administered routine care for the two groups, who were blind to group allocations. Likewise, a blinded nurse collected all data in the two groups. Also, to reduce prejudice, acupressure was performed by an assistant different from the data collector. Additionally, the data analyzer was unaware of the codes assigned to the study groups.

### Data collection

The study tool comprised a demographic questionnaire, St. Mary’s Hospital Sleep Questionnaire (SMHSQ), Spielberger’s State-Trait Anxiety Inventory for State Anxiety (STAI-S), and the adverse events form. A blinded research assistant completed all parts.

The demographic questionnaire included items about the patient’s demographic and clinical characteristics, completed before the random allocation by interviewing the patients and extracting information from their clinical records. The research team fellows designed this questionnaire; then, its qualitative validity (i.e., face and content) was confirmed by ten panelists.

To measure the patients’ sleep quality, the Persian version of SMHSQ was filled out before the random assignment (as the baseline) and after three nights of acupressure, on the morning of the fourth day (as the post-test). This self-completed questionnaire consists of eight items and aims to measure the duration and mental quality of a hospitalized patient’s sleep during the previous night. The response of each item is according to a Likert scale varying from one to four, leading to a total score of 8–32 [43]. Lower scores mean a higher quality of sleep and vice versa [44]. Recent Iranian studies have applied the Persian version of SMHSQ among burn patients [17, 26, 28]. Based on the data from 72 recruited patients, Cronbach’s alpha coefficient was 0.83 in the current survey.

To measure the patients’ anxiety, the Persian version of STAI-S was completed simultaneously with the SMHSQ. The STAI-S evaluates the subjective feelings of tension, worry, nervousness, apprehension, and autonomic nervous system activation. This self-completed inventory consisted of 20 items on a four-point Likert scale running from one (i.e., not at all) to four (i.e., very much). Hence, the total score varies from 20 to 80, with higher obtained scores describing more elevated levels of anxiety [45]. The psychometric properties of the Persian version of STAI-S have been confirmed by previous investigations [46]. Also, it is a commonly used tool by Iranian scholars [47], and its reliability was reported as acceptable among patients with burns [17, 28]. In the present study, the coefficient of Cronbach’s alpha for this tool was 0.91.

The occurrence of any potential adverse effects (i.e., itching and irritation) was monitored by daily examination of nursing staff. Also, all patients were requested to report the negative impacts of the interventions.

### Intervention process

The routine care for the two groups was similar and presented by blinded nursing assistants at a time other than night (i.e., 10:00 p.m. to 7:00 a.m.) to not disturb the patients’ night sleep. Based on the care protocol of the recruitment center, all patients routinely received daily wound debridement and dressing. Patients also received intravenous midazolam and morphine when needed, mainly before their dressing changes, based on the physicians’ assessment. Moreover, all patients were urged to follow standard sleep considerations (i.e., decreasing ambient lights and noises). Besides, all patients received a 30-minute face-to-face instruction from the principal investigator on taking care of their burn injuries using uncomplicated and understandable sentences. Moreover, all the patients were oriented to prevent self-acupressure outside of the acupressurist-delivered intervention and avoid the usage of any other medications than the prescribed drugs.

**Fig. 1:**
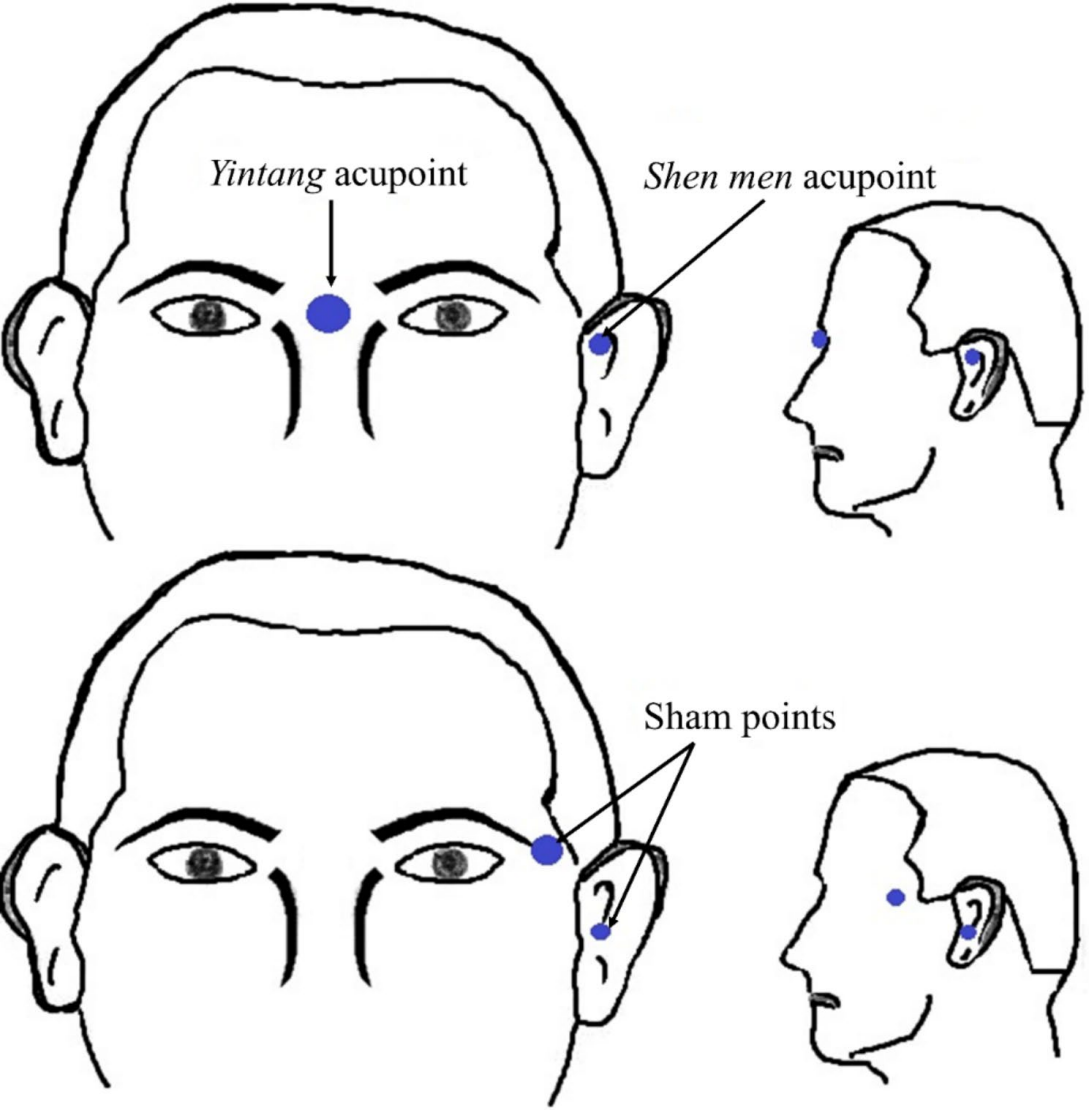
Location of acupressure points in the experimental group (i.e., *Yintang* and *Shen men*) and sham group

The patients in the experimental arm received acupressure in the real locations, whereas those in the control arm received acupressure in the sham areas. We selected *Yintang* and *Shen men* acupoints based on the suggestion of the assistant acupressurist. The first acupoint locates between the eyebrows and the root of the nasal, and the second one is in the upper partition of the non-dominant ear cavity. The sham areas for *Yintang* and *Shen men* acupoints were the outer corner of the left eyebrow and the entry of the non-dominant ear cavity, respectively, which are known as points that have no sedative effects **(**Fig. 1). To determine the feasibility of these locations, the research team performed a pilot study among burn patients with similar characteristics to current trial participants from August 2020 to March 2021. The findings showed the safety of real and sham locations described above, as none of the patients experienced adverse effects [42].

The acupressure for the two groups was applied once a day for three consecutive days before night sleep by the same acupressurist (i.e., a nurse with a certificate for acupressure administration). Before all acupressure sessions, the acupressurist thoroughly cleaned her hands and disinfected each acupressure location with an alcohol-based pad; subsequently, patients rested comfortably in bed and received acupressure in the sham or real points. To apply acupressure, first, the patients determined their non-dominant ear according to whether they were left- or right-handed; after that, they received the acupressure based on their study group. In the experimental group, the *Yintang* acupoint was massaged by the thumb via a simple superficial rotational technique for 10 min, averaging 20–25 times per minute (total frequency of 200–250 times). In the control group, the outer corner of the left eyebrow was massaged with a similar procedure, frequency, and duration to the experimental group. During the 10-minute *Yintang* acupressure, a small plastic pin was placed on the non-dominant ear cavity to pressure the *Shen men* acupoint. Similarly, in the control group, a small plastic pin was fixed on the entrance of the non-dominant ear cavity for 10 min when the outer corner of the left eyebrow was massaged. All the acupressure details (e.g., locations, technique, duration, and frequency) were adopted from the pilot trial [42]; nevertheless, based on the acupressurist’s suggestion, the interventions were administered for three days instead of two consecutive days performed in the pilot trial.

### Ethical considerations

The trial was supported by the Local Research Ethics Committee of Arak University of Medical Sciences, Arak, Iran (ethical license code: IR.ARAKMU.REC.1399.338, trial registry code: IRCT20130424013110N13, 19/03/2021, https://www.irct.ir/trial/55076). Prior to the trial commencement, the research methods and objectives were presented to all eligible participants. Furthermore, they were ensured that their information would be kept confidential and used solely for research purposes. Finally, if participants volunteered to partake in the trial, they signed written informed consent.

### Statistical methods

The sample size was computed with the following formula proposed to determine the reasonable sample size for seeing a difference between the means of two samples. Considering β = 0.20, α = 0.05, an anticipated effect size (d) = 0.70, and group ratio (r) = 1, an optimal sample size of approximately 33 subjects per group was calculated. However, we selected 36 patients in each group, considering 10% sample attrition.

$$\begin{gathered}{\text{n}} \geqslant \left( {\frac{{1 + r}}{r}} \right)\frac{{{{\left( {{z_{1 - \frac{\alpha }{2}}} + {z_{1 - \beta }}} \right)}^2}}}{{{d^2}}} + \frac{{{{\left( {{z_{1 - \frac{\alpha }{2}}}} \right)}^2}}}{{2\left( {1 + r} \right)}} = \left( {\frac{{1 + 1}}{1}} \right)\frac{{{{\left( {1.96 + 0.84} \right)}^2}}}{{{{0.70}^2}}} \hfill \\+ \frac{{{{1.96}^2}}}{{2\left( {1 + 1} \right)}} = 32.9 \hfill \\ \end{gathered}$$

All data were analyzed utilizing the Statistical Package for Social Sciences software (SPSS, SPSS Inc., USA). The homogeneity of study groups for demographic and clinical information was addressed with the Chi-square test (or Fisher’s exact test) and the independent samples *t*-test for nominal and continuous variables, respectively. Also, to compare the means of SMHSQ and STAI-S scores between and within groups, the independent samples *t*-test and paired samples *t*-test were employed, respectively. Additionally, the independent samples *t*-test was utilized to evaluate the groups regarding the mean changes in SMHSQ and STAI-S scores from the baseline to the end of the trial. A *P*-value < 0.05 was assumed statistically significant.

## Results

### Study population characteristics

**Fig. 2:**
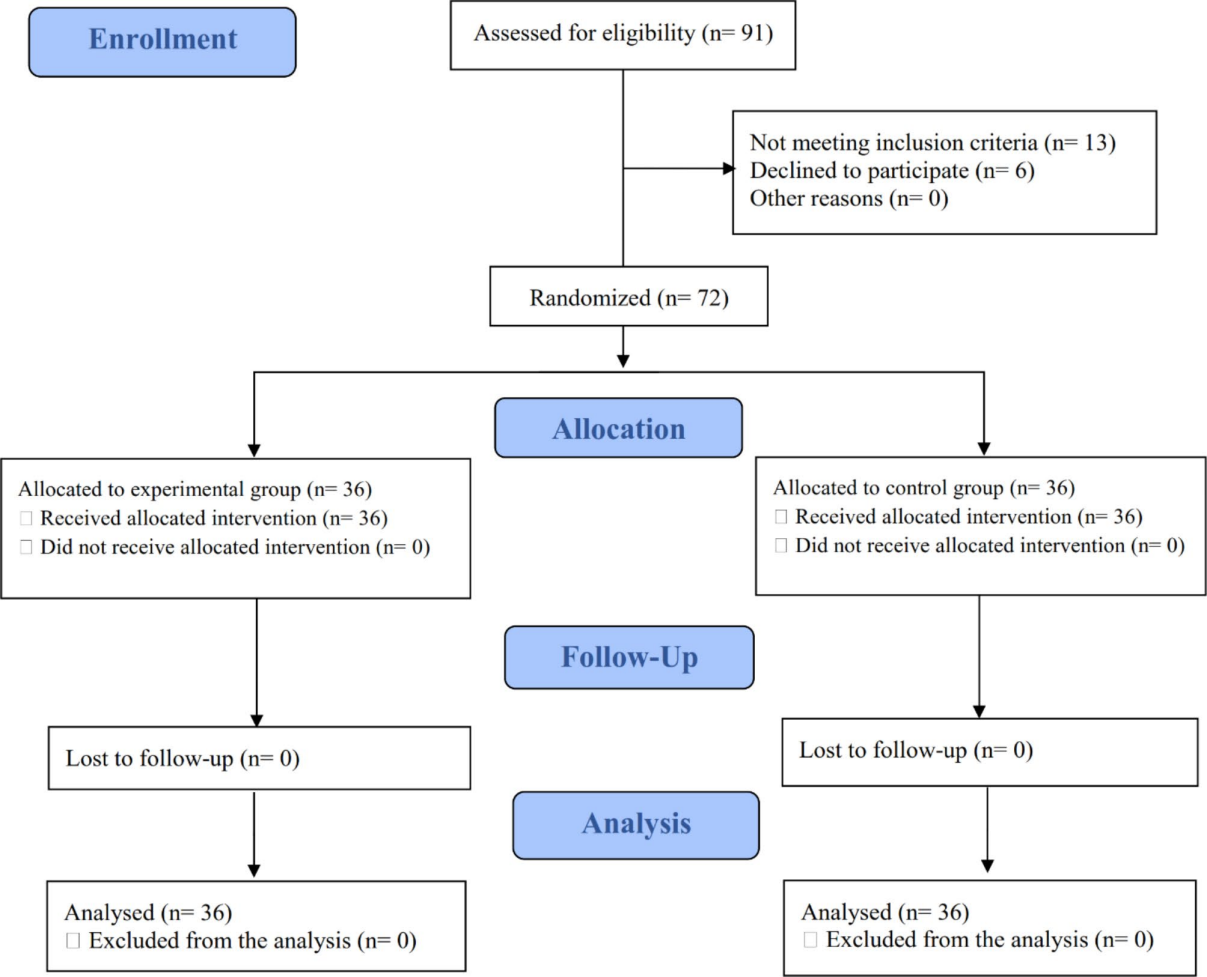
CONSORT flow diagram of the study participants

Ninety-one patients were examined for being eligible to partake in this trial. Nonetheless, six declined to participate, and 13 did not meet some inclusion criteria. All 72 randomized patients ended the trial and were incorporated into the final analysis (Fig. 2). Participants’ demographical and clinical features are summarized in Table 1.

**Table 1:** Demographic and clinical characteristics of the study groups

		Experimental group (*n* = 36)	Control group (*n* = 36)	Between-group *P*-value
**Qualitative variables**		N (%)	N (%)	
**Gender**	Male	19 (52.8)	19 (52.8)	1.00 ^*^
	Female	17 (47.2)	17 (47.2)	
**Marital Status**	Single	3 (8.3)	5 (13.9)	0.905 ^**^
	Married	29 (80.6)	28 (77.8)	
	Divorced	1 (2.8)	1 (2.8)	
	Widow	3 (8.3)	2 (5.5)	
**Job status**	Jobless	1 (2.8)	1 (2.8)	0.949 ^**^
	Housewife	1 (2.8)	1 (2.8)	
	Employed	22 (61.0)	20 (55.5)	
	Retired	12 (33.4)	14 (38.9)	
**Educational level**	Primary	7 (19.4)	6 (16.7)	0.300 ^*^
	High School	18 (50.0)	24 (66.6)	
	Academic	11 (30.6)	6 (16.7)	
**Quantitative variables**		**Mean ± SD**	**Mean ± SD**	
**Age (years)**		39.36 ± 8.47	41.17 ± 7.33	0.336 ^***^
**Burn extent (%TBSA)**		35.92 ± 8.38	37.92 ± 11.19	0.394 ^***^

TBSA: Total body surface area

**Note**: Quantitative variables have been expressed as mean ± standard deviation (SD), while qualitative variables have been presented as number (percentage).

^*^ Chi-square test

^**^ Fisher’s exact test

^***^ Independent samples *t*-test

### Main outcomes

At the baseline, the independent samples *t*-test did not display any significant between-group differences in the mean scores of SMHSQ and STAI-S (*P =* 0.864, *P =* 0.360). Nevertheless, this test confirmed that the post-test mean scores of SMHSQ and STAI-S in the experimental arm were significantly lower than in the control arm *(P <* 0.001 in two cases). Besides, considering the results of the paired samples *t*-test, the mean score of SMHSQ decreased significantly at the post-test compared to the baseline in the experimental arm *(P <* 0.001); however, in the control arm, a significant increase was found (*P =* 0.006). Also, the mean score of STAI-S was significantly reduced at the end of interventions as compared to the baseline in the experimental arm (*P <* 0.001). In contrast, in the control arm, this difference was not meaningful (*P =* 0.128) (Table 2).

**Table 2:** Comparison of sleep quality and anxiety in the study groups

Variables		Experimental group (*n* = 36)	Control group (*n* = 36)	Between-group *P*-value^*^
		Mean ± SD	Mean ± SD	
**Sleep quality** ^**1**^	Before intervention	23.27 ± 1.99	23.19 ± 2.10	0.864
	After intervention	14.75 ± 2.08	24.61 ± 1.97	< 0.001
	Within-group *P*-value^**^	< 0.001	0.006	
**Anxiety** ^**2**^	Before intervention	59.30 ± 5.62	60.50 ± 5.38	0.360
	After intervention	35.11 ± 3.80	58.72 ± 3.55	< 0.001
	Within-group *P*-value^**^	< 0.001	0.128	

All values have been expressed as mean ± standard deviation (SD).

^1^ St. Mary’s Hospital Sleep Questionnaire: the total score ranges from 8–32, lower scores indicating higher sleep quality.

^2^ Spielberger’s State-Trait Anxiety Inventory for State Anxiety: the total score ranges from 20–80, lower scores representing lower anxiety levels.

^*^ Independent samples *t*-test

^**^ Paired samples *t*-test

**Fig. 3:**
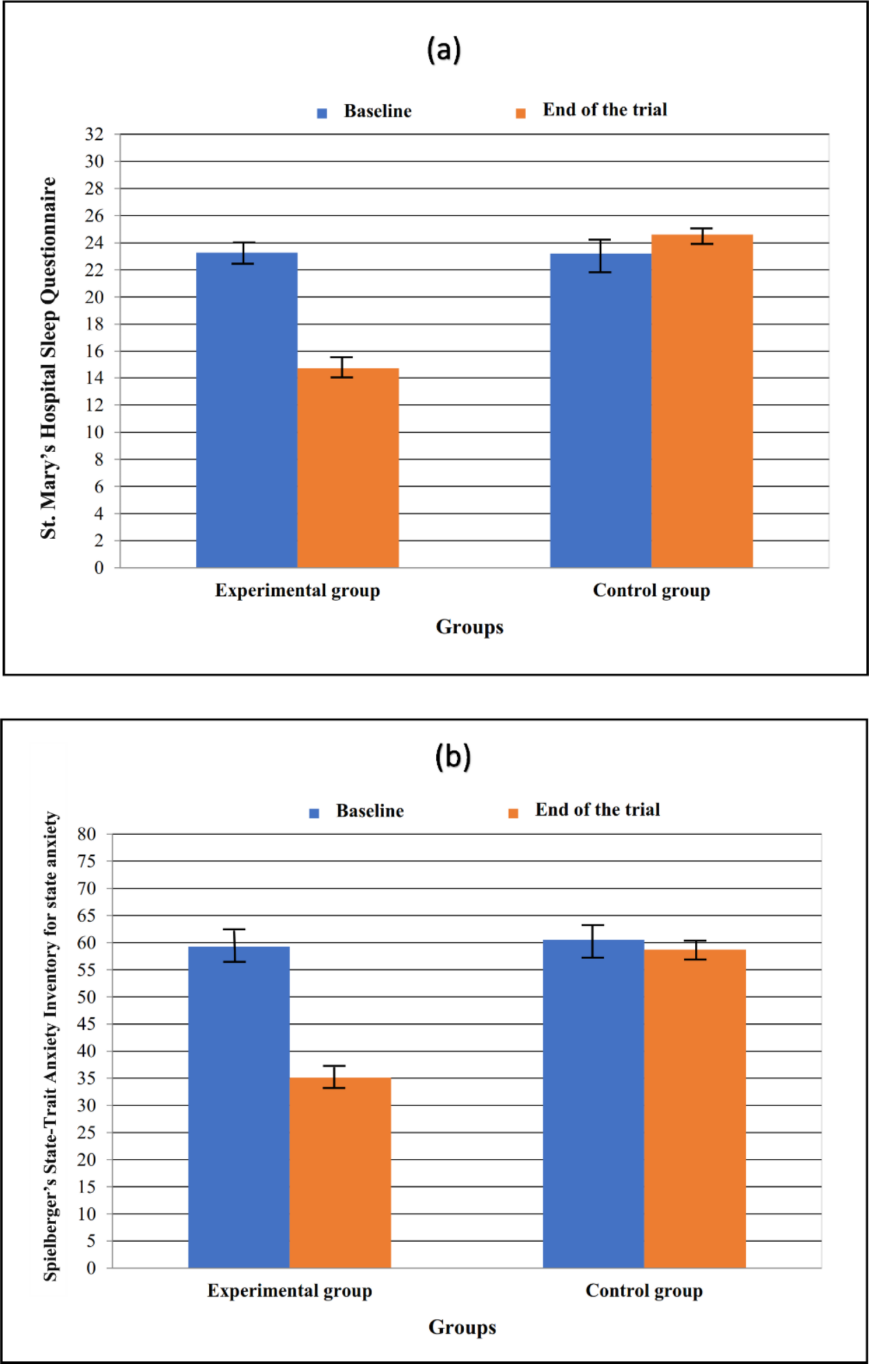
Changes in scores of sleep quality **(a)** and anxiety **(b)** from the baseline to the end of the trial in the study groups

The independent samples *t*-test also showed a significant between-group difference in the mean change of SMHSQ score from the baseline to the post-test (˗8.52 ± 2.51 vs. 1.41 ± 2.91, *P <* 0.001). Such finding was also found for the mean difference of STAI-S score (˗24.19 ± 7.53 vs. ˗1.77 ± 9.83, *P <* 0.001). As illustrated in Fig. 3, the decrease in mean changes was considerably more in the experimental arm than in the control arm.

### Adverse effects

None of the participants encountered any adverse effects related to the interventions.

## Discussion

Despite advances in the pharmaceutical management of burn-induced anxiety and sleeplessness, related medications are associated with different side effects. Hence, the potential impacts of various complementary/alternative non-pharmacological practices have been increasingly examined in recent years for this purpose [17, 28]. Accordingly, we investigated the potential efficacy of 10-minute acupressure in *Yintang and Shen men* acupoints in enhancing the quality of sleep and alleviating anxiety levels among burn patients. Findings revealed that the SMHSQ and STAI-S scores were remarkably lower in the real acupressure group at the trial’s end than in the sham acupressure group. Also, the reduction in mean changes of SMHSQ and STAI-S scores from baseline to the end of the trial was significantly more in patients who received the real acupressure. These findings imply that acupressure could effectively reduce anxiety and boost the quality of sleep among burn sufferers.

As far as we know, no trial has previously addressed the impacts of acupressure on the quality of sleep and anxiety among patients with burns. However, consistent with the current study, some studies found the beneficial effect of acupressure on other health outcomes of burn patients. In a randomized controlled trial, Harorani et al. reported that post-dressing pain was relieved more in Iranian burn victims who had received 10-minute acupressure in *Yintang* and *Shen men* acupoints for two consecutive days compared to those who had undergone the sham acupressure with the same duration and technique [42]. Also, in a one-group pretest-posttest trial, Jung Ok showed a significant effect of a six-month skin rehabilitation massage therapy (i.e., manipulation of the skin and underlying tissues with light palm stroking, acupressure, and an occlusive dressing) on reducing stress levels of Korean burn survivors [48].

Consistent with our findings, some trials reported that acupressure could decline both sleep disturbances and anxiety levels among patients undergoing cardiac surgery [49], nursing home residents [50], and nursing students [51]. Contrary to the present study, some studies documented conflicting findings on the anxiety-alleviating and sleep-enhancing effects of acupressure. In an integrative review of nine studies on acupressure interventions for elders, Hmwe et al. found that acupressure could augment sleep quality, but inconclusive findings were obtained for the effect of this method on anxiety [29]. Similarly, in a single-masked trial, Bang and Park revealed that acupressure increased the score of the Sleep Quality Scale more than the sham acupressure at 14 days post-surgery; however, no substantial between-group difference was seen in the STAI-S score [30]. Perhaps the lack of consistency in our results and the mentioned findings is related to the differences in participants’ clinical conditions, acupressure protocols, and follow-up periods. We administered acupressure in *Yintang and Shen men* acupoints for 10 min before night sleep for three consecutive nights among burn patients aged 18–55. However, in the discussed trial, auricular acupressure was applied in five acupoints (i.e., *Shen men*, *Occiput*, *Sympathy*, *Heart*, and *Anterior lobe*) six days a week for two consecutive weeks among patients undergoing cardiac surgery aged 40–71 years. Accordingly, a shorter duration of the intervention in the present study, alongside administrating acupressure in a lower number of acupoints and recruiting patients with lower ages, might lead to better effects in the current study. Additionally, the authors of the mentioned study attributed their unexpected findings to a higher level of anxiety among candidates for cardiac surgeries.

The current study’s findings also substantiate the available evidence evaluating the efficacy of acupressure administration on either sleep quality or anxiety of patients with other disorders [33–37]. However, a trial executed by Agarwal et al. showed the acupressure efficacy in diminishing the preoperative score of the Visual Stress Scale immediately at the end of acupressure application for 10 min, while its impacts were not durable 30 min after acupressure administration [52]. The results of the mentioned trial are compatible with our results, but we did not consider the sustainability of the acupressure effect on anxiety.

Although the exact mechanism of the impacts of acupressure on the quality of sleep and anxiety levels in burn survivors has not been specified, the means through which this method can affect these outcomes in other disorders have been presented. According to the philosophy of acupressure, this complementary approach could induce sleep, calmness, and psychological health by regulating neuro-hormonal responses [53]. Stimulation of acupoints can modulate the autonomic nervous system by decreasing sympathetic actions and raising parasympathetic activities, which leads to declining sleep problems, anxiety levels, and the requirement for sedatives [30]. Acupoint stimulation also affects neurotransmitters and hormones, which play substantial roles in sleep regulation (i.e., melatonin, serotonin, acetylcholine, adrenocorticotrophic hormone, and endorphins), leading to augmented sleep quality [29]. Based on the classical theory of traditional Chinese medicine, the simulation of *Yintang* acupoint can reduce insomnia by regulating melatonin. Besides, this point is known for removing feelings of restlessness and helps with overall emotional well-being [31]. Also, *Shen men* is considered an excellent point for reducing pain and mental disorders (e.g., anxiety, stress, and depression), stabilizing emotion, and regulating anti-inflammatory activity [54].

### Study implications

Burn patients often experience anxiety and sleep disturbances, hindering their recovery. Nurses play an essential role in enhancing sleep quality and controlling the anxiety of these patients [26]. Hence, they must pursue appropriate related nursing interventions. Based on the present research findings, acupressure as a low-cost nursing intervention, probably without side effects, can be potentially valuable for boosting the quality of sleep and decreasing anxiety among patients with burns. Accordingly, to control and treat these patients’ sleep problems and anxiety, acupressure-based interventions could be applied as complementary modalities along with routine care and treatments. Also, acupressure seems suitable for patients with limited access to clinical centers, especially in low-income countries where burn-related injuries are reported higher, because they can learn and use it easily. Therefore, nurses and other health professionals should train and encourage patients with burn-induced sleep disturbances and anxiety to undertake acupressure by themselves.

### Study strengths and limitations

This trial is the initial attempt to examine the acupressure effectiveness on burn-induced sleep disturbances and anxiety. A qualified nurse performed acupressure in two study groups, which could be a vigorous aspect of this study. Also, we used a sham comparator to resemble the acupressurist’s support and communication level with patients in the control group, identical to those in the experimental group, which could be another substantial aspect of this study. However, due to the nature of the intervention, it was impossible to completely blind the patients to acupressure treatment, which may have influenced the reported results due to different expectations or perceptions of patients in the experimental group compared to those in the control group. Likewise, we performed a short-term follow-up and only evaluated the outcomes at the end of the three-day trial. Hence, whether the observed effects persisted over a long period is unclear. Besides, we measured only subjective sleep quality and anxiety using self-report tools, which might be influenced by participants’ interpretation and response bias, as well as present a biased view of the whole experience of these outcomes. Finally, the study was conducted in one center with a small sample size; therefore, the results might only be representative of some of the community.

## Conclusion

Administrating short-term acupressure, which included three 10-minute sessions, was effective and safe in improving sleep quality and relieving anxiety among hospitalized patients suffering from burn injuries. Yet, forthcoming trials are suggested to consider the mid-to-long-term impacts of acupressure on the study outcomes with more samples to identify the sustainability of the findings and provide more reliable evidence on the safety and efficacy of this method in alleviating sleep disturbances and anxiety among burn victims. Also, for a trustworthy analysis of sleep quality and anxiety, using objective or physiological research tools in addition to subjective measures may be helpful.

## Data Availability

Data and materials are available by contacting the corresponding author.

## Abbreviations

SMHSQ: St. Mary’s Hospital Sleep Questionnaire
SPSS: Statistical package for social sciences
STAI-S: Spielberger’s State-Trait Anxiety Inventory for State Anxiety
